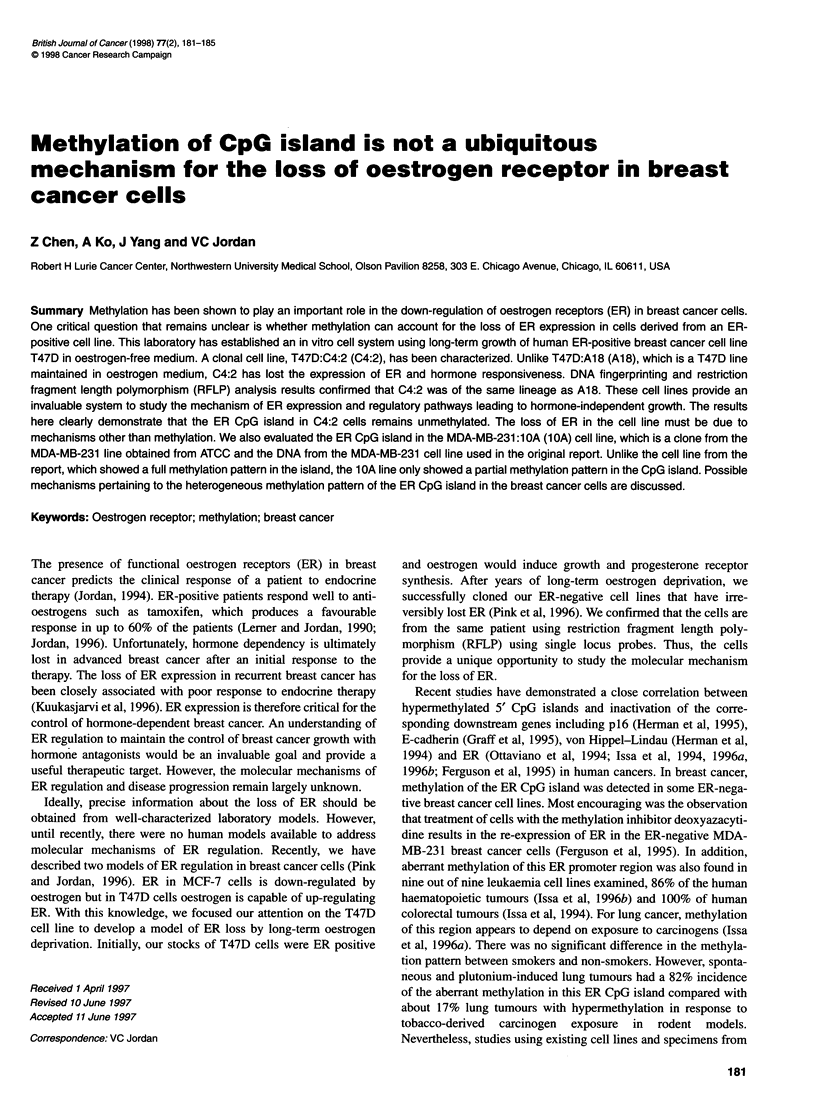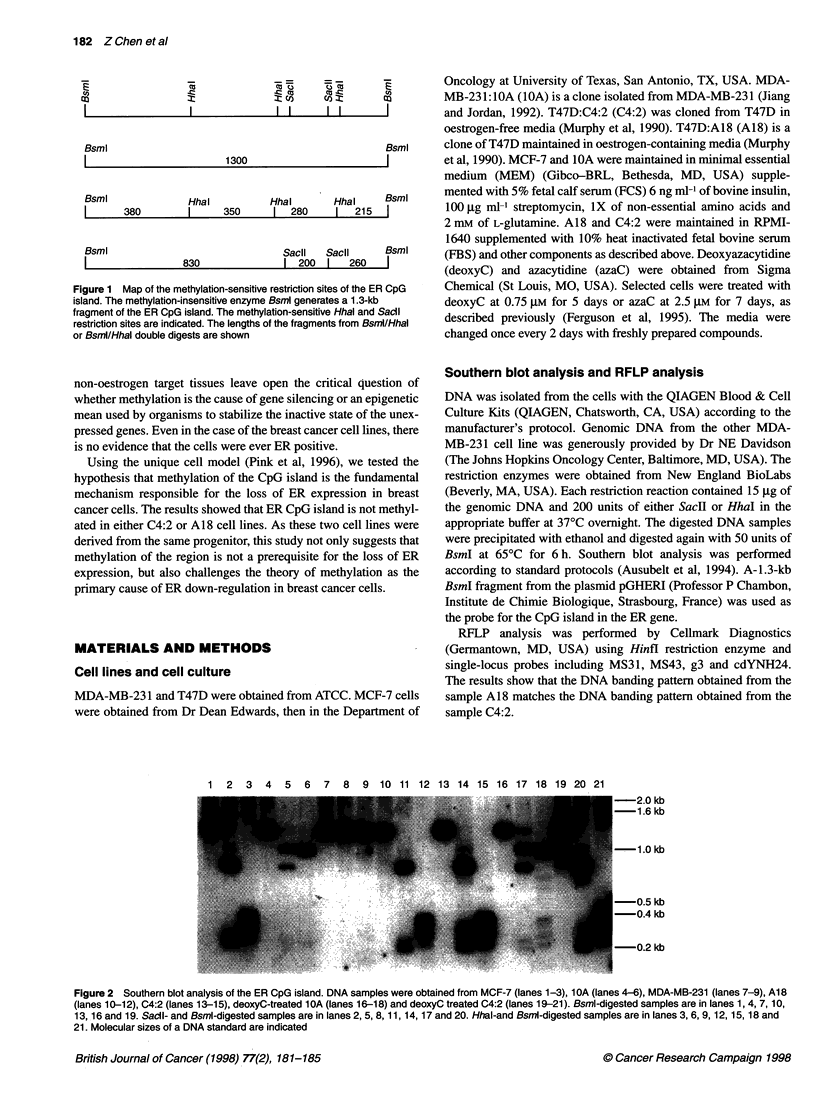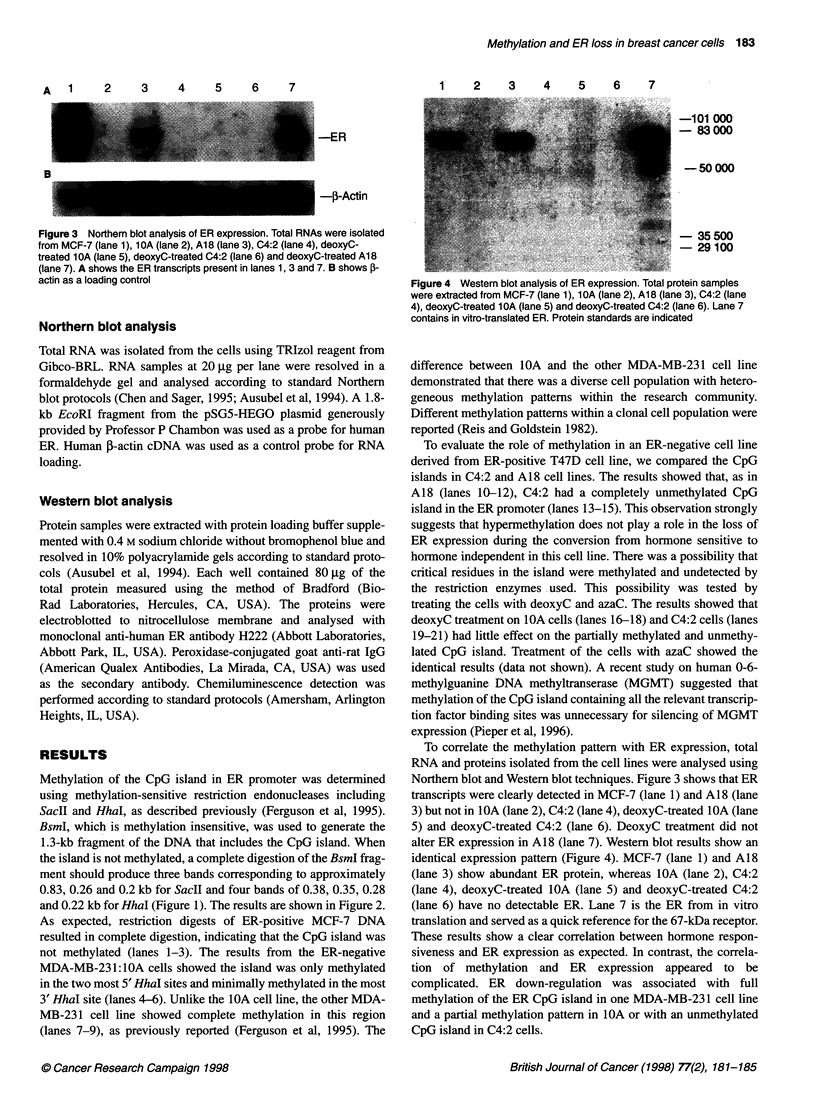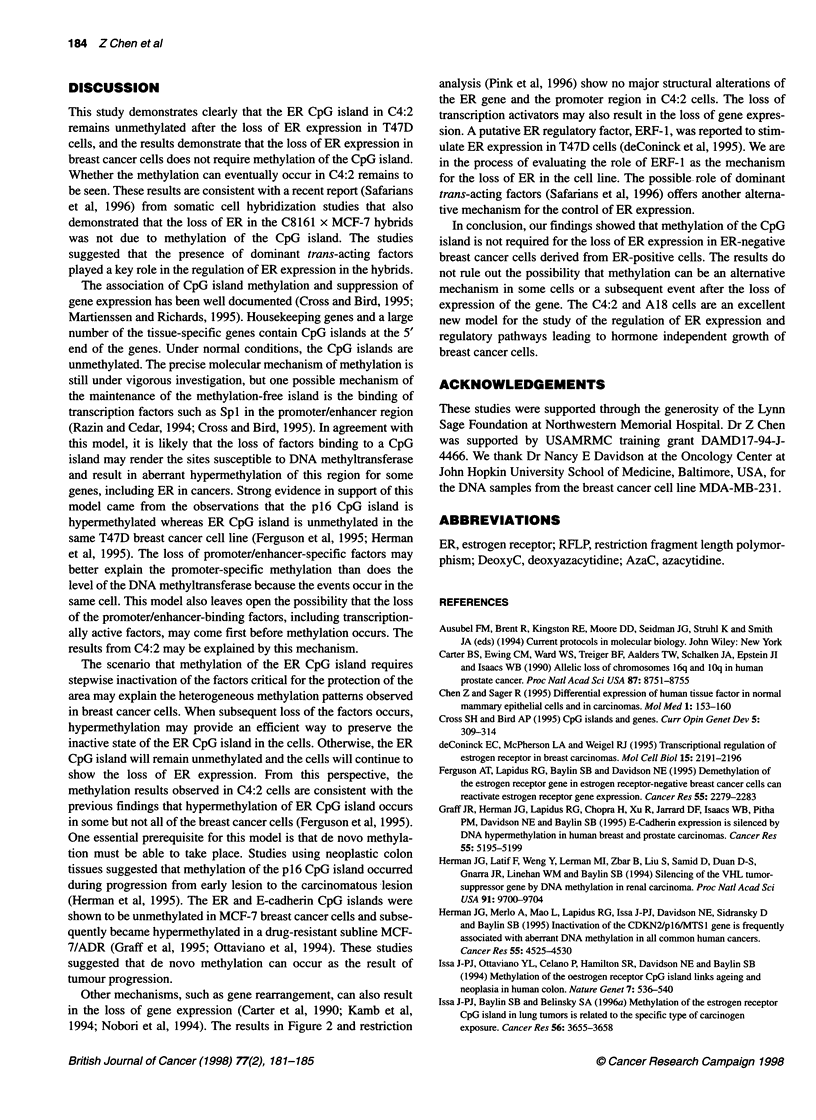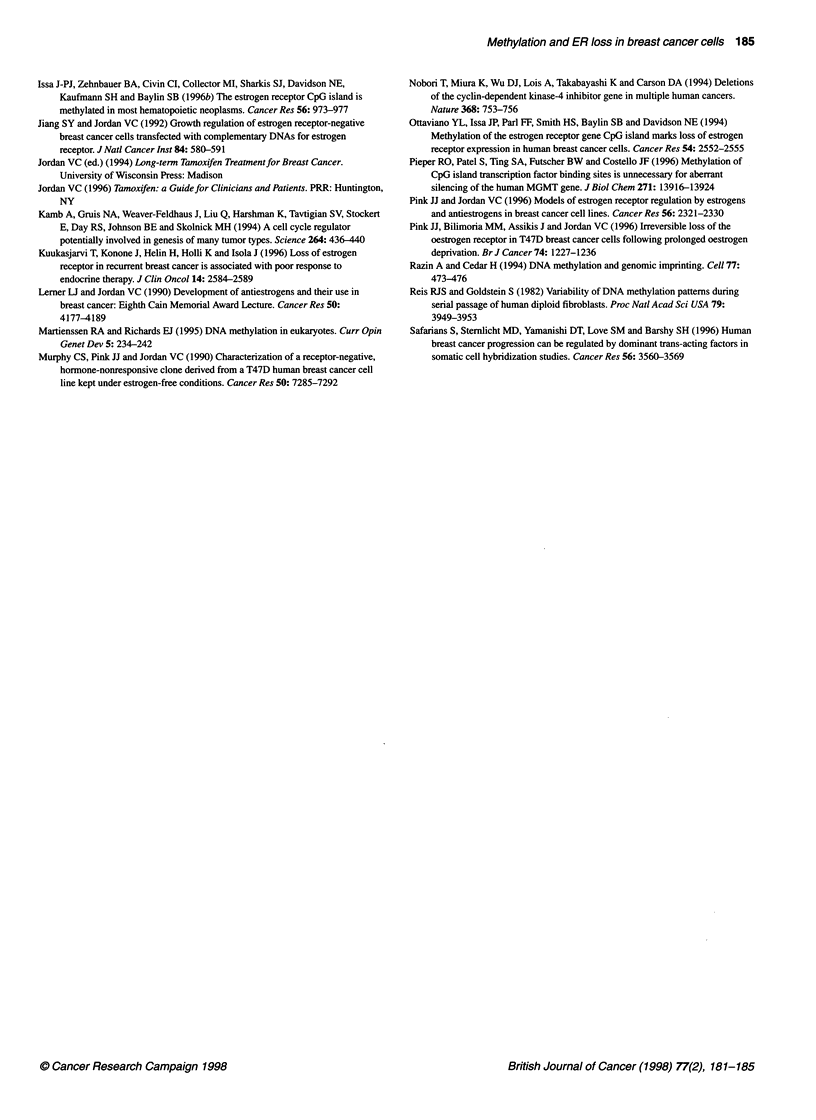# Methylation of CpG island is not a ubiquitous mechanism for the loss of oestrogen receptor in breast cancer cells.

**DOI:** 10.1038/bjc.1998.31

**Published:** 1998

**Authors:** Z. Chen, A. Ko, J. Yang, V. C. Jordan

**Affiliations:** Robert H Lurie Cancer Center, Northwestern University Medical School, Chicago, IL 60611, USA.

## Abstract

**Images:**


British Journal of Cancer (1998) 77(2), 181–185
© 1998 Cancer Research Campaign

### Methylation of CpG island is not a ubiquitous mechanism for the loss of oestrogen receptor in breast cancer cells

**Z Chen, A Ko, J Yang and VC Jordan**

Robert H Lurie Cancer Center, Northwestern University Medical School, Olson Pavilion 8258, 303 E. Chicago Avenue, Chicago, IL 60611, USA

**Summary** Methylation has been shown to play an important role in the down-regulation of oestrogen receptors (ER) in breast cancer cells. One critical question that remains unclear is whether methylation can account for the loss of ER expression in cells derived from an ER-positive cell line. This laboratory has established an in vitro cell system using long-term growth of human ER-positive breast cancer cell line T47D in oestrogen-free medium. A clonal cell line, T47D:C4:2 (C4:2), has been characterized. Unlike T47D:A18 (A18), which is a T47D line maintained in oestrogen medium, C4:2 has lost the expression of ER and hormone responsiveness. DNA fingerprinting and restriction fragment length polymorphism (RFLP) analysis results confirmed that C4:2 was of the same lineage as A18. These cell lines provide an invaluable system to study the mechanism of ER expression and regulatory pathways leading to hormone-independent growth. The results here clearly demonstrate that the ER CpG island in C4:2 cells remains unmethylated. The loss of ER in the cell line must be due to mechanisms other than methylation. We also evaluated the ER CpG island in the MDA-MB-231:10A (10A) cell line, which is a clone from the MDA-MB-231 line obtained from ATCC and the DNA from the MDA-MB-231 cell line used in the original report. Unlike the cell line from the report, which showed a full methylation pattern in the island, the 10A line only showed a partial methylation pattern in the CpG island. Possible mechanisms pertaining to the heterogeneous methylation pattern of the ER CpG island in the breast cancer cells are discussed.

**Keywords:** Oestrogen receptor; methylation; breast cancer

The presence of functional oestrogen receptors (ER) in breast cancer predicts the clinical response of a patient to endocrine therapy (Jordan, 1994). ER-positive patients respond well to anti-oestrogens such as tamoxifen, which produces a favourable response in up to 60% of the patients (Lerner and Jordan, 1990; Jordan, 1996). Unfortunately, hormone dependency is ultimately lost in advanced breast cancer after an initial response to the therapy. The loss of ER expression in recurrent breast cancer has been closely associated with poor response to endocrine therapy (Kuukasjarvi et al, 1996). ER expression is therefore critical for the control of hormone-dependent breast cancer. An understanding of ER regulation to maintain the control of breast cancer growth with hormone antagonists would be an invaluable goal and provide a useful therapeutic target. However, the molecular mechanisms of ER regulation and disease progression remain largely unknown.

Ideally, precise information about the loss of ER should be obtained from well-characterized laboratory models. However, until recently, there were no human models available to address molecular mechanisms of ER regulation. Recently, we have described two models of ER regulation in breast cancer cells (Pink and Jordan, 1996). ER in MCF-7 cells is down-regulated by oestrogen but in T47D cells oestrogen is capable of up-regulating ER. With this knowledge, we focused our attention on the T47D cell line to develop a model of ER loss by long-term oestrogen deprivation. Initially, our stocks of T47D cells were ER positive and oestrogen would induce growth and progesterone receptor synthesis. After years of long-term oestrogen deprivation, we successfully cloned our ER-negative cell lines that have irreversibly lost ER (Pink et al, 1996). We confirmed that the cells are from the same patient using restriction fragment length polymorphism (RFLP) using single locus probes. Thus, the cells provide a unique opportunity to study the molecular mechanism for the loss of ER.

Recent studies have demonstrated a close correlation between hypermethylated 5′ CpG islands and inactivation of the corresponding downstream genes including p16 (Herman et al, 1995), E-cadherin (Graff et al, 1995), von Hippel–Lindau (Herman et al, 1994) and ER (Ottaviano et al, 1994; Issa et al, 1994, 1996*a*, 1996*b*; Ferguson et al, 1995) in human cancers. In breast cancer, methylation of the ER CpG island was detected in some ER-negative breast cancer cell lines. Most encouraging was the observation that treatment of cells with the methylation inhibitor deoxyazacytidine results in the re-expression of ER in the ER-negative MDA-MB-231 breast cancer cells (Ferguson et al, 1995). In addition, aberrant methylation of this ER promoter region was also found in nine out of nine leukaemia cell lines examined, 86% of the human haematopoietic tumours (Issa et al, 1996*b*) and 100% of human colorectal tumours (Issa et al, 1994). For lung cancer, methylation of this region appears to depend on exposure to carcinogens (Issa et al, 1996*a*). There was no significant difference in the methylation pattern between smokers and non-smokers. However, spontaneous and plutonium-induced lung tumours had a 82% incidence of the aberrant methylation in this ER CpG island compared with about 17% lung tumours with hypermethylation in response to tobacco-derived carcinogen exposure in rodent models. Nevertheless, studies using existing cell lines and specimens from

*Received 1 April 1997*
*Revised 10 June 1997*
*Accepted 11 June 1997*

*Correspondence:* VC Jordan

**Figure 1** Map of the methylation-sensitive restriction sites of the ER CpG island. The methylation-insensitive enzyme *BsmI* generates a 1.3-kb fragment of the ER CpG island. The methylation-sensitive *HhaI* and *SacII* restriction sites are indicated. The lengths of the fragments from *BsmI/HhaI* or *BsmI/HhaI* double digests are shown non-oestrogen target tissues leave open the critical question of whether methylation is the cause of gene silencing or an epigenetic mean used by organisms to stabilize the inactive state of the unexpressed genes. Even in the case of the breast cancer cell lines, there is no evidence that the cells were ever ER positive.

Using the unique cell model (Pink et al, 1996), we tested the hypothesis that methylation of the CpG island is the fundamental mechanism responsible for the loss of ER expression in breast cancer cells. The results showed that ER CpG island is not methylated in either C4:2 or A18 cell lines. As these two cell lines were derived from the same progenitor, this study not only suggests that methylation of the region is not a prerequisite for the loss of ER expression, but also challenges the theory of methylation as the primary cause of ER down-regulation in breast cancer cells.

### MATERIALS AND METHODS

### Cell lines and cell culture

MDA-MB-231 and T47D were obtained from ATCC. MCF-7 cells were obtained from Dr Dean Edwards, then in the Department of Oncology at University of Texas, San Antonio, TX, USA. MDA-MB-231:10A (10A) is a clone isolated from MDA-MB-231 (Jiang and Jordan, 1992). T47D:C4:2 (C4:2) was cloned from T47D in oestrogen-free media (Murphy et al, 1990). T47D:A18 (A18) is a clone of T47D maintained in oestrogen-containing media (Murphy et al, 1990). MCF-7 and 10A were maintained in minimal essential medium (MEM) (Gibco–BRL, Bethesda, MD, USA) supplemented with 5% fetal calf serum (FCS) 6 ng $ml^{-1}$ of bovine insulin, 100 μg $ml^{-1}$ streptomycin, 1X of non-essential amino acids and 2 mM of L-glutamine. A18 and C4:2 were maintained in RPMI-1640 supplemented with 10% heat inactivated fetal bovine serum (FBS) and other components as described above. Deoxyazacytidine (deoxyC) and azacytidine (azaC) were obtained from Sigma Chemical (St Louis, MO, USA). Selected cells were treated with deoxyC at 0.75 μM for 5 days or azaC at 2.5 μM for 7 days, as described previously (Ferguson et al, 1995). The media were changed once every 2 days with freshly prepared compounds.

### Southern blot analysis and RFLP analysis

DNA was isolated from the cells with the QIAGEN Blood & Cell Culture Kits (QIAGEN, Chatsworth, CA, USA) according to the manufacturer's protocol. Genomic DNA from the other MDA-MB-231 cell line was generously provided by Dr NE Davidson (The Johns Hopkins Oncology Center, Baltimore, MD, USA). The restriction enzymes were obtained from New England BioLabs (Beverly, MA, USA). Each restriction reaction contained 15 μg of the genomic DNA and 200 units of either *Sac*II or *Hha*I in the appropriate buffer at 37°C overnight. The digested DNA samples were precipitated with ethanol and digested again with 50 units of *Bsm*I at 65°C for 6 h. Southern blot analysis was performed according to standard protocols (Ausubelt et al, 1994). A-1.3-kb *Bsm*I fragment from the plasmid pGHERI (Professor P Chambon, Institute de Chimie Biologique, Strasbourg, France) was used as the probe for the CpG island in the ER gene.

RFLP analysis was performed by Cellmark Diagnostics (Germantown, MD, USA) using *Hinf*I restriction enzyme and single-locus probes including MS31, MS43, g3 and cdYNH24. The results show that the DNA banding pattern obtained from the sample A18 matches the DNA banding pattern obtained from the sample C4:2.

**Figure 2** Southern blot analysis of the ER CpG island. DNA samples were obtained from MCF-7 (lanes 1–3), 10A (lanes 4–6), MDA-MB-231 (lanes 7–9), A18 (lanes 10–12), C4:2 (lanes 13–15), deoxyC-treated 10A (lanes 16–18) and deoxyC treated C4:2 (lanes 19–21). *BsmI*-digested samples are in lanes 1, 4, 7, 10, 13, 16 and 19. *SacII*- and *BsmI*-digested samples are in lanes 2, 5, 8, 11, 14, 17 and 20. *HhaI*-and *BsmI*-digested samples are in lanes 3, 6, 9, 12, 15, 18 and 21. Molecular sizes of a DNA standard are indicated

© Cancer Research Campaign 1998

**Figure 3** Northern blot analysis of ER expression. Total RNAs were isolated from MCF-7 (lane 1), 10A (lane 2), A18 (lane 3), C4:2 (lane 4), deoxyC-treated 10A (lane 5), deoxyC-treated C4:2 (lane 6) and deoxyC-treated A18 (lane 7). **A** shows the ER transcripts present in lanes 1, 3 and 7. **B** shows β-actin as a loading control

**Figure 4** Western blot analysis of ER expression. Total protein samples were extracted from MCF-7 (lane 1), 10A (lane 2), A18 (lane 3), C4:2 (lane 4), deoxyC-treated 10A (lane 5) and deoxyC-treated C4:2 (lane 6). Lane 7 contains in vitro-translated ER. Protein standards are indicated

### Northern blot analysis

Total RNA was isolated from the cells using TRIzol reagent from Gibco-BRL. RNA samples at 20 μg per lane were resolved in a formaldehyde gel and analysed according to standard Northern blot protocols (Chen and Sager, 1995; Ausubel et al, 1994). A 1.8-kb *Eco*RI fragment from the pSG5-HEGO plasmid generously provided by Professor P Chambon was used as a probe for human ER. Human β-actin cDNA was used as a control probe for RNA loading.

### Western blot analysis

Protein samples were extracted with protein loading buffer supplemented with 0.4 M sodium chloride without bromophenol blue and resolved in 10% polyacrylamide gels according to standard protocols (Ausubel et al, 1994). Each well contained 80 μg of the total protein measured using the method of Bradford (Bio-Rad Laboratories, Hercules, CA, USA). The proteins were electroblotted to nitrocellulose membrane and analysed with monoclonal anti-human ER antibody H222 (Abbott Laboratories, Abbott Park, IL, USA). Peroxidase-conjugated goat anti-rat IgG (American Qualex Antibodies, La Mirada, CA, USA) was used as the secondary antibody. Chemiluminescence detection was performed according to standard protocols (Amersham, Arlington Heights, IL, USA).

### RESULTS

Methylation of the CpG island in ER promoter was determined using methylation-sensitive restriction endonucleases including *Sac*II and *Hha*I, as described previously (Ferguson et al, 1995). *Bsm*I, which is methylation insensitive, was used to generate the 1.3-kb fragment of the DNA that includes the CpG island. When the island is not methylated, a complete digestion of the *Bsm*I fragment should produce three bands corresponding to approximately 0.83, 0.26 and 0.2 kb for *Sac*II and four bands of 0.38, 0.35, 0.28 and 0.22 kb for *Hha*I (Figure 1). The results are shown in Figure 2. As expected, restriction digests of ER-positive MCF-7 DNA resulted in complete digestion, indicating that the CpG island was not methylated (lanes 1–3). The results from the ER-negative MDA-MB-231:10A cells showed the island was only methylated in the two most 5′ *Hha*I sites and minimally methylated in the most 3′ *Hha*I site (lanes 4–6). Unlike the 10A cell line, the other MDA-MB-231 cell line showed complete methylation in this region (lanes 7–9), as previously reported (Ferguson et al, 1995). The difference between 10A and the other MDA-MB-231 cell line demonstrated that there was a diverse cell population with heterogeneous methylation patterns within the research community. Different methylation patterns within a clonal cell population were reported (Reis and Goldstein 1982).

To evaluate the role of methylation in an ER-negative cell line derived from ER-positive T47D cell line, we compared the CpG islands in C4:2 and A18 cell lines. The results showed that, as in A18 (lanes 10–12), C4:2 had a completely unmethylated CpG island in the ER promoter (lanes 13–15). This observation strongly suggests that hypermethylation does not play a role in the loss of ER expression during the conversion from hormone sensitive to hormone independent in this cell line. There was a possibility that critical residues in the island were methylated and undetected by the restriction enzymes used. This possibility was tested by treating the cells with deoxyC and azaC. The results showed that deoxyC treatment on 10A cells (lanes 16–18) and C4:2 cells (lanes 19–21) had little effect on the partially methylated and unmethylated CpG island. Treatment of the cells with azaC showed the identical results (data not shown). A recent study on human 0-6-methylguanine DNA methyltranserase (MGMT) suggested that methylation of the CpG island containing all the relevant transcription factor binding sites was unnecessary for silencing of MGMT expression (Pieper et al, 1996).

To correlate the methylation pattern with ER expression, total RNA and proteins isolated from the cell lines were analysed using Northern blot and Western blot techniques. Figure 3 shows that ER transcripts were clearly detected in MCF-7 (lane 1) and A18 (lane 3) but not in 10A (lane 2), C4:2 (lane 4), deoxyC-treated 10A (lane 5) and deoxyC-treated C4:2 (lane 6). DeoxyC treatment did not alter ER expression in A18 (lane 7). Western blot results show an identical expression pattern (Figure 4). MCF-7 (lane 1) and A18 (lane 3) show abundant ER protein, whereas 10A (lane 2), C4:2 (lane 4), deoxyC-treated 10A (lane 5) and deoxyC-treated C4:2 (lane 6) have no detectable ER. Lane 7 is the ER from in vitro translation and served as a quick reference for the 67-kDa receptor. These results show a clear correlation between hormone responsiveness and ER expression as expected. In contrast, the correlation of methylation and ER expression appeared to be complicated. ER down-regulation was associated with full methylation of the ER CpG island in one MDA-MB-231 cell line and a partial methylation pattern in 10A or with an unmethylated CpG island in C4:2 cells.

© Cancer Research Campaign 1998

### DISCUSSION

This study demonstrates clearly that the ER CpG island in C4:2 remains unmethylated after the loss of ER expression in T47D cells, and the results demonstrate that the loss of ER expression in breast cancer cells does not require methylation of the CpG island. Whether the methylation can eventually occur in C4:2 remains to be seen. These results are consistent with a recent report (Safarians et al, 1996) from somatic cell hybridization studies that also demonstrated that the loss of ER in the C8161 × MCF-7 hybrids was not due to methylation of the CpG island. The studies suggested that the presence of dominant *trans*-acting factors played a key role in the regulation of ER expression in the hybrids.

The association of CpG island methylation and suppression of gene expression has been well documented (Cross and Bird, 1995; Martienssen and Richards, 1995). Housekeeping genes and a large number of the tissue-specific genes contain CpG islands at the 5′ end of the genes. Under normal conditions, the CpG islands are unmethylated. The precise molecular mechanism of methylation is still under vigorous investigation, but one possible mechanism of the maintenance of the methylation-free island is the binding of transcription factors such as Sp1 in the promoter/enhancer region (Razin and Cedar, 1994; Cross and Bird, 1995). In agreement with this model, it is likely that the loss of factors binding to a CpG island may render the sites susceptible to DNA methyltransferase and result in aberrant hypermethylation of this region for some genes, including ER in cancers. Strong evidence in support of this model came from the observations that the p16 CpG island is hypermethylated whereas ER CpG island is unmethylated in the same T47D breast cancer cell line (Ferguson et al, 1995; Herman et al, 1995). The loss of promoter/enhancer-specific factors may better explain the promoter-specific methylation than does the level of the DNA methyltransferase because the events occur in the same cell. This model also leaves open the possibility that the loss of the promoter/enhancer-binding factors, including transcriptionally active factors, may come first before methylation occurs. The results from C4:2 may be explained by this mechanism.

The scenario that methylation of the ER CpG island requires stepwise inactivation of the factors critical for the protection of the area may explain the heterogeneous methylation patterns observed in breast cancer cells. When subsequent loss of the factors occurs, hypermethylation may provide an efficient way to preserve the inactive state of the ER CpG island in the cells. Otherwise, the ER CpG island will remain unmethylated and the cells will continue to show the loss of ER expression. From this perspective, the methylation results observed in C4:2 cells are consistent with the previous findings that hypermethylation of ER CpG island occurs in some but not all of the breast cancer cells (Ferguson et al, 1995). One essential prerequisite for this model is that de novo methylation must be able to take place. Studies using neoplastic colon tissues suggested that methylation of the p16 CpG island occurred during progression from early lesion to the carcinomatous lesion (Herman et al, 1995). The ER and E-cadherin CpG islands were shown to be unmethylated in MCF-7 breast cancer cells and subsequently became hypermethylated in a drug-resistant subline MCF-7/ADR (Graff et al, 1995; Ottaviano et al, 1994). These studies suggested that de novo methylation can occur as the result of tumour progression.

Other mechanisms, such as gene rearrangement, can also result in the loss of gene expression (Carter et al, 1990; Kamb et al, 1994; Nobori et al, 1994). The results in Figure 2 and restriction analysis (Pink et al, 1996) show no major structural alterations of the ER gene and the promoter region in C4:2 cells. The loss of transcription activators may also result in the loss of gene expression. A putative ER regulatory factor, ERF-1, was reported to stimulate ER expression in T47D cells (deConinck et al, 1995). We are in the process of evaluating the role of ERF-1 as the mechanism for the loss of ER in the cell line. The possible role of dominant *trans*-acting factors (Safarians et al, 1996) offers another alternative mechanism for the control of ER expression.

In conclusion, our findings showed that methylation of the CpG island is not required for the loss of ER expression in ER-negative breast cancer cells derived from ER-positive cells. The results do not rule out the possibility that methylation can be an alternative mechanism in some cells or a subsequent event after the loss of expression of the gene. The C4:2 and A18 cells are an excellent new model for the study of the regulation of ER expression and regulatory pathways leading to hormone independent growth of breast cancer cells.

### ACKNOWLEDGEMENTS

These studies were supported through the generosity of the Lynn Sage Foundation at Northwestern Memorial Hospital. Dr Z Chen was supported by USAMRMC training grant DAMD17-94-J-4466. We thank Dr Nancy E Davidson at the Oncology Center at John Hopkin University School of Medicine, Baltimore, USA, for the DNA samples from the breast cancer cell line MDA-MB-231.

### ABBREVIATIONS

ER, estrogen receptor; RFLP, restriction fragment length polymorphism; DeoxyC, deoxyazacytidine; AzaC, azacytidine.

### REFERENCES

Ausubel FM, Brent R, Kingston RE, Moore DD, Seidman JG, Struhl K and Smith JA (eds) (1994) Current protocols in molecular biology. John Wiley: New York

Carter BS, Ewing CM, Ward WS, Treiger BF, Aalders TW, Schalken JA, Epstein JI and Isaacs WB (1990) Allelic loss of chromosomes 16q and 10q in human prostate cancer. *Proc Natl Acad Sci USA* **87:** 8751–8755

Chen Z and Sager R (1995) Differential expression of human tissue factor in normal mammary epithelial cells and in carcinomas. *Mol Med* **1:** 153–160

Cross SH and Bird AP (1995) CpG islands and genes. *Curr Opin Genet Dev* **5:** 309–314

deConinck EC, McPherson LA and Weigel RJ (1995) Transcriptional regulation of estrogen receptor in breast carcinomas. *Mol Cell Biol* **15:** 2191–2196

Ferguson AT, Lapidus RG, Baylin SB and Davidson NE (1995) Demethylation of the estrogen receptor gene in estrogen receptor-negative breast cancer cells can reactivate estrogen receptor gene expression. *Cancer Res* **55:** 2279–2283

Graff JR, Herman JG, Lapidus RG, Chopra H, Xu R, Jarrard DF, Isaacs WB, Pitha PM, Davidson NE and Baylin SB (1995) E-Cadherin expression is silenced by DNA hypermethylation in human breast and prostate carcinomas. *Cancer Res* **55:** 5195–5199

Herman JG, Latif F, Weng Y, Lerman MI, Zbar B, Liu S, Samid D, Duan D-S, Gnarra JR, Linehan WM and Baylin SB (1994) Silencing of the VHL tumor-suppressor gene by DNA methylation in renal carcinoma. *Proc Natl Acad Sci USA* **91:** 9700–9704

Herman JG, Merlo A, Mao L, Lapidus RG, Issa J-PJ, Davidson NE, Sidransky D and Baylin SB (1995) Inactivation of the CDKN2/p16/MTS1 gene is frequently associated with aberrant DNA methylation in all common human cancers. *Cancer Res* **55:** 4525–4530

Issa J-PJ, Ottaviano YL, Celano P, Hamilton SR, Davidson NE and Baylin SB (1994) Methylation of the oestrogen receptor CpG island links ageing and neoplasia in human colon. *Nature Genet* **7:** 536–540

Issa J-PJ, Baylin SB and Belinsky SA (1996*a*) Methylation of the estrogen receptor CpG island in lung tumors is related to the specific type of carcinogen exposure. *Cancer Res* **56:** 3655–3658

© Cancer Research Campaign 1998

Issa J-PJ, Zehnbauer BA, Civin CI, Collector MI, Sharkis SJ, Davidson NE, Kaufmann SH and Baylin SB (1996*b*) The estrogen receptor CpG island is methylated in most hematopoietic neoplasms. *Cancer Res* **56:** 973–977

Jiang SY and Jordan VC (1992) Growth regulation of estrogen receptor-negative breast cancer cells transfected with complementary DNAs for estrogen receptor. *J Natl Cancer Inst* **84:** 580–591

Jordan VC (ed.) (1994) *Long-term Tamoxifen Treatment for Breast Cancer*. University of Wisconsin Press: Madison

Jordan VC (1996) *Tamoxifen: a Guide for Clinicians and Patients*. PRR: Huntington, NY

Kamb A, Gruis NA, Weaver-Feldhaus J, Liu Q, Harshman K, Tavtigian SV, Stockert E, Day RS, Johnson BE and Skolnick MH (1994) A cell cycle regulator potentially involved in genesis of many tumor types. *Science* **264:** 436–440

Kuukasjarvi T, Konone J, Helin H, Holli K and Isola J (1996) Loss of estrogen receptor in recurrent breast cancer is associated with poor response to endocrine therapy. *J Clin Oncol* **14:** 2584–2589

Lerner LJ and Jordan VC (1990) Development of antiestrogens and their use in breast cancer: Eighth Cain Memorial Award Lecture. *Cancer Res* **50:** 4177–4189

Martienssen RA and Richards EJ (1995) DNA methylation in eukaryotes. *Curr Opin Genet Dev* **5:** 234–242

Murphy CS, Pink JJ and Jordan VC (1990) Characterization of a receptor-negative, hormone-nonresponsive clone derived from a T47D human breast cancer cell line kept under estrogen-free conditions. *Cancer Res* **50:** 7285–7292

Nobori T, Miura K, Wu DJ, Lois A, Takabayashi K and Carson DA (1994) Deletions of the cyclin-dependent kinase-4 inhibitor gene in multiple human cancers. *Nature* **368:** 753–756

Ottaviano YL, Issa JP, Parl FF, Smith HS, Baylin SB and Davidson NE (1994) Methylation of the estrogen receptor gene CpG island marks loss of estrogen receptor expression in human breast cancer cells. *Cancer Res* **54:** 2552–2555

Pieper RO, Patel S, Ting SA, Futscher BW and Costello JF (1996) Methylation of CpG island transcription factor binding sites is unnecessary for aberrant silencing of the human MGMT gene. *J Biol Chem* **271:** 13916–13924

Pink JJ and Jordan VC (1996) Models of estrogen receptor regulation by estrogens and antiestrogens in breast cancer cell lines. *Cancer Res* **56:** 2321–2330

Pink JJ, Bilimoria MM, Assikis J and Jordan VC (1996) Irreversible loss of the oestrogen receptor in T47D breast cancer cells following prolonged oestrogen deprivation. *Br J Cancer* **74:** 1227–1236

Razin A and Cedar H (1994) DNA methylation and genomic imprinting. *Cell* **77:** 473–476

Reis RJS and Goldstein S (1982) Variability of DNA methylation patterns during serial passage of human diploid fibroblasts. *Proc Natl Acad Sci USA* **79:** 3949–3953

Safarians S, Sternlicht MD, Yamanishi DT, Love SM and Barshy SH (1996) Human breast cancer progression can be regulated by dominant trans-acting factors in somatic cell hybridization studies. *Cancer Res* **56:** 3560–3569

© Cancer Research Campaign 1998